# An overlap of Alport syndrome and rheumatoid arthritis in a patient and literature review

**DOI:** 10.1186/s12882-019-1462-3

**Published:** 2019-07-23

**Authors:** Xiaofei Tang, Qiuling Ding, Dong Xu, Songtao Yang, Yuefei Xiao, Jian Liu

**Affiliations:** 1 Department of Nephrology and Rheumatology, Aerospace Center Hospital, Beijing, China; 2 Department of Rheumatism and Immunology, Peking Union Medical College Hospital, Beijing, China

**Keywords:** Alport syndrome, rheumatoid arthritis, type IV collagen, microhematuria, *COL4A5*

## Abstract

**Background:**

Alport syndrome is a rare genetic kidney disease, and rheumatoid arthritis as a common autoimmune disease also causes renal lesions in addition to arthritis. The overlap of them has rarely been reported.

**Case presentation:**

A 44-year-old man had a history of multi-joint swelling and pain for more than half a year. His laboratory data with double positive for rheumatoid factor and anticitrullinated protein antibodies further supported the diagnosis of early rheumatoid arthritis. His previous medical history including progressive hearing loss for several years and microhematuria for one year attracted our attention. Renal biopsy showed thin basement membrane nephropathy and lymphocytes infiltration of interstitium. To make a precise diagnosis, targeted Next Generation Sequencing (NGS) of an inherited renal disease panel including Alport syndrome genes was performed, which revealed the missense mutation in *COL4A5* (c.1351 T > C, p.Cys451Arg). Further in silico analyses predicted that the p. Cys451Arg mutation is functionally “damaging”, so the diagnosis of Alport syndrome was finally proved. The patient has been receiving the treatment of total glucosides of paeony and leflunomide for rheumatoid arthritis, and Cozaar 50 mg for the protection of kidney so far. During the 10-months follow-up, swelling and tenderness of the joints in this patient had been generally relieved, with no obvious improvement in microhematuria and a slight increase in proteinuria.

**Conclusion:**

we reported an adult man with the coexistence of rheumatoid arthritis and Alport syndrome with the missense mutation in *COL4A5* (c.1351 T > C, p.Cys451Arg). Whether the overlap of them is occasional or has a common pathophysiological mechanism is still unclear.

## Background

Alport syndrome is a relatively rare genetic disease which characterized hematuria and/or proteinuria, progressive renal failure, hearing loss, and ocular abnormalities [1]. It is related to mutations in the genes encoding one of three chains, α3, α4, and α5 of type IV collagen, the main component of basement membranes, expressed in the glomerular basement membrane (GBM), cochlea, retina, lens capsule, and cornea. 85% of patients have the X-linked forms with *COL4A5* mutations, and the others have mutations in *COL4A3* or *COL4A4* with autosomal forms [1, 2].

Rheumatoid arthritis (RA) is an autoimmune disease in which persistent inflammatory responses occur in multiple synovial joints and extra-articular organs [3]. Renal involvement such as urinary abnormalities and renal dysfunction can be observed in RA patients, which may be mainly mediated by nephrotoxic effects of numerous drugs such as NSAIDs and DMARDs used in RA treatment and less by RA itself according to existing literature [3, 4].

It has been rarely reported that Alport syndrome and RA occur simultaneously or successively in the same patient. Because some clinical manifestations of them such as hematuria and ocular abnormalities may overlap, and it may be meaningful to discuss the pathogenesis of both diseases. We reported a case of an Alport syndrome patient overlap with RA and discussed the possible connection between them by literature review.

## Case presentation

A 44-year-old male, Chinese businessman, mainly for multi-joints swelling and pain for more than half a year, was admitted to Department of Nephrology and Rheumatology in our hospital on January 9, 2018. Six months before admission, he presented successively multiple joint arthralgia and mild swelling, mainly involving shoulder joints, the PIP joints of both hands, the second and third of MCP joints of both hands, double wrist joints, with morning stiffness for a few minutes. In the course of the disease, he had no symptoms of dry eyes, blurred vision, mouth ulcer, rash and hair loss, etc. He accepted “three oxygen therapy” in Horqin First Hospital, which made swelling and pain of above joints partially relieved. However, his right shoulder joint pain got worse one week before admission, and he was admitted to our hospital. His previous medical history included progressive hearing loss for several years and microhematuria for one year by a physical examination. His father died of colorectal ulcer. His mother is relatively healthy without hearing loss and obvious kidney diseases, and she never had a urine test. His daughter also had a history of microhematuria found by a medical examination for enrollment one year ago. On physical examination, his vital signs including blood pressure, body temperature, respiration rate and pulse rate were within normal range, and no abnormalities were found in lungs, heart and abdomen. The PIP joints of both hands, the second and third of MCP joints of both hands, and his right shoulder joint had mild tenderness with no obvious swelling.

Laboratory data was as follows: blood routine was normal; urinalysis revealed hematuria (3+) with 100% of deformed red blood cells by phase difference microscopic examination and microalbuminuria (0.101 g/24 h); blood chemistry showed normal function of liver, serum creatinine within normal range (106 μmol/L) and moderately increased level of blood uric acid (526 μmol/L); inflammatory markers including C-reactive protein and ESR were normal; serological studies revealed an elevated level of RF (46.0 IU/ml), a dramatically elevated level of ACPA (>250RU/ml), normal levels of immunoglobulin and complement; ANA and ANCA were negative; serum hepatitis B surface antigen and anti-hepatitis C virus antibody were negative. The X-ray of both hands was normal. There were no abnormalities on chest computed tomography and electrocardiogram. Renal ultrasound showed diffuse changes in double renal parenchyma and double renal multiple cysts with partial cystic wall calcification. The results of hearing test suggested binaural sensorineural deafness, and no abnormalities were found in the eye examination.

**Fig. 1:**
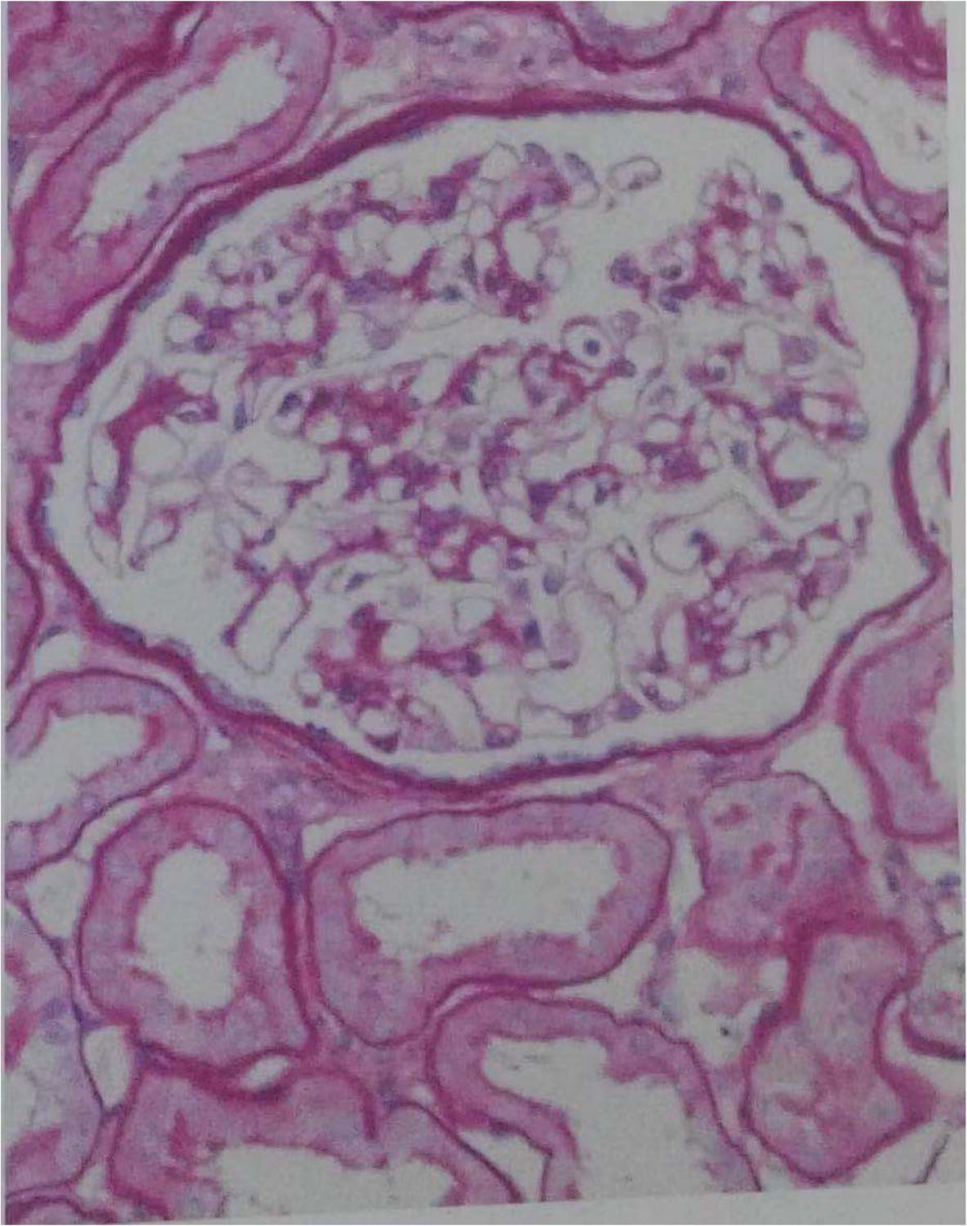
Light microscopy in renal biopsy specimen. Mild glomerular lesions with chronic renal tubular interstitial lesion (PAS stain× 200)

**Fig. 2:**
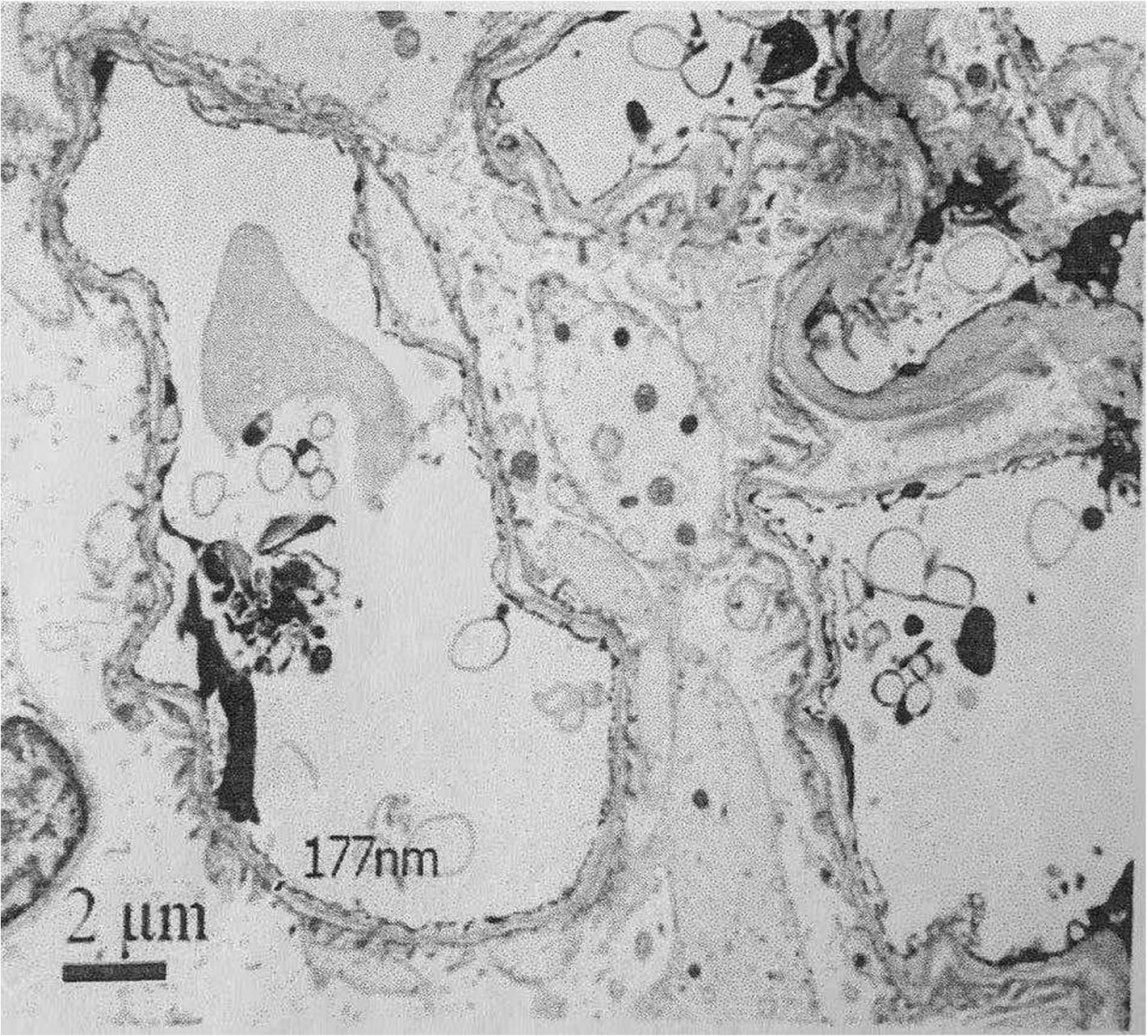
Electron microscopy in renal biopsy specimen. Diffuse thinning of GBM (GBM, 130-200 nm), epithelial foot process segmental fusion, no dense deposit, edema and lymphocytes infiltration of interstitium

The patient with swelling and tenderness of muti-joints over six weeks, double-positive for RF and ACPA, according to 2010 ACR/EULAR classification criteria for RA [5], was definitely diagnosed with early RA. However, a history of microhematuria could not be completely explained by RA, as microhematuria occurred before RA. To further clarify the cause of hematuria, a renal biopsy was performed on January 18, 2018. Light microscopy showed glomerular minimal change with chronic tubulointerstitial disease, and immunofluorescence microscopy showed mild stainings for IgM(+) and IgA(±), with others negative (Fig. 1). Electron microscopy revealed thin GBM (130-200 nm), epithelial foot process segmental fusion, no dense deposit, with edema and lymphocytes infiltration of interstitium (Fig. 2). The diagnosis of electron was TBMN with tubular interstitiallesion (Fig. 2). Combining with microhematuria, hearing loss and TBMN, we highly suspected that he had a very rare disease for Alport syndrome. Mutational Analysis of Alport syndrome genes (COL4A3, COL4A4 and COL4A5) by Beijing Genomics Institute revealed the mutation in *COL4A5* (c.1351 T > C, p.Cys451Arg, hemizygous, missense mutation) encoding α5 of type IV collagen. In silico analyses using Database of Non-synonymous Functional Predictions (dbNSFP) predicted that p.Cys451Arg is functionally “tolerated”(0.07, scores less than 0.05 are classified as “damaging”) by Sorting Intolerant From Tolerant (SIFT), “possibly damaging” (0.603, Polymorphism Phenotyping 2 HumVar), “probably damaging”(0.971, Polymorphism Phenotyping 2 HumDiv), “probably damaging”(0.963, Mutation Taster), and.

“deleterious” by Likelihood Ratio Test. Above results further proved the diagnosis of X-linked Alport syndrome.

The patient has been regularly receiving the treatment of total glucosides of paeony and leflunomide for RA, and Cozaar 50 mg for the protection of kidney so far. During the 10-months follow-up, swelling and tenderness of the joints in this patient had been generally relieved, but there was no obvious improvement in microhematuria and a slight increase in proteinuria.

## Discussion and conclusions

Renal lesions such as urinary abnormalities and renal dysfunction in RA patients can be often observed. The mechanism of renal involvement of RA may be complicated, consisting of glomerular damage due, mainly, to secondary amyloidosis and membranous nephropathy (related to gold salts, D-penicillamine), and also tubular damage (due to NSAIDs]) [6, 7]. However, an active immunological process may participate in the development of immune complex-type renal lesions in some RA patients [8]. Skrifvars reported that 15 of 20 patient with RA by renal biopsy had glomerular deposits containing IgG and/or C3, IgM, and IgA [8]. Therefore, renal lesions might occur during the prolonged and active course of RA.

The present patient with the persistence of polyarthritis over six weeks, positive RF, and a high titer of ACPA (>250RU/ml), was definitely diagnosed with the early RA according to 2010 ACR/EULAR classification criteria for RA [5]. The history of hematuria and hearing loss before arthritis in this patient attracted our attention, which associated with RA or other kidney diseases such as Alport syndrome or IgA nephropathy should be further examined. Subsequent pathological diagnosis of TBMN by electron microscopy in renal biopsy specimen and the *COL4A5* gene mutation in this patient supported the diagnosis of Alport syndrome. Although we did not do immunohistochemistry for expression of type IV collagen alpha 5 chain protein in GBM, in silico analyses of c.1351 T > C revealed that p. Cys451Arg was probably damaging and finally lead to the remodeling of GBM. Therefore, the appearance of microhematuria before RA in this patient was mainly the result of Alport syndrome, and had no correlation with RA. As we all know, in addition to microhematuria, ocular abnormalities often occur in Alport syndrome, and these symptoms may be misdiagnosed as the extra-articular manifestations of RA. Therefore, clinicians should be aware of using “dualism” for the explanation of condition in any case.

Classical X-linked Alport syndrome is characterized by early End Stage Kidney Disease (ESKD), neurosensory deafness and ocular defects, with characteristic ultrastructural features (diffuse thickening with splitting of the lamina densa into multiple interweaving strands) [9]. Inactivating nonsense, frameshift mutations and large deletions are the usual causes [9, 10]. However, a small number of X-linked Alport syndrome patients, due to milder, missense mutations, such as G624D and P628L, may only exhibit microhematuria, TBMN, mild chronic renal failure (CRF) or late onset ESKD [9, 10]. The present patient, a 44-year-old male, only exhibited mild microhematuria, less proteinuria and TBMN, which might correlate with his mild phenotype (c.1351 T > C, p.Cys451Arg, hemizygous, missense mutation). His daughter inherited the mutant gene (c.1351 T > C, heterozygous) with only mild microhematuria. It was unclear whether his mother had microhematuria, because she had never done any urine tests. However, she was over 70 years old and apparently healthy without deafness, blurred vision and manifestations of CRF. Therefore, his mild clinical manifestations and phenotype might indicate a better prognosis for him and his family members.

The overlap of Alport syndrome and RA in one patient has rarely been reported. Whether the overlap of them in this patient is occasional or has a common pathophysiological mechanism is unclear. As we all know, synovitis is the main pathological change of RA.

Despite an absence of a basement membrane similar to that surrounding blood vessels in normal synovial lining composed of macrophage-like Type A and fibroblast-like type B lining cells, some basement membrane components such as type IV collagen can be found in its intercellular substance [11]. In addition, the assessment by immunohistochemical stainings shows that all type IV collagen alpha-chains can be found in the vascular basement membrane in the synovial lining layer [12]. The presence of autoantibodies to collagen IV has been found in some rheumatologic diseases. For example, 20% of children with juvenile RA, 52% of adults with RA, and 60% of patients with systemic lupus erythematosus (SLE) had antibodies to one or more of the four collagen antigens [13]. Recently, Gudmann NS reported that type IV collagen remodelling was associated with disease activity and radiographic progression in RA [12]. Therefore, it was possible that the production of autoantibodies induced by abnormal type-IV collagen combining with other environmental factors triggered the onset of RA in present patient.

This case highlights RA patients with microhematuria and/or proteinuria should attract the serious attention, and a renal biopsy is necessary to differentiate RA-related renal lesion or other kidney diseases. TBMN and X-linked, *COL4A5* missense mutation in this patient might indicate a relatively better prognosis. Although the overlap of Alport syndrome and RA in present case may be occasional, abnormal type IV collagen might participate in the pathogenesis of RA, which needs more related case reports and studies to prove in future.

## Data Availability

The datasets used and/or analyzed during the current study available from the corresponding author on reasonable request.

## Abbreviations

ACPA: anticitrullinated protein antibodies
ACR/EULAR: American college of rheumatology/European league against rheumatology
ANA: anti-nuclear antibodies
ANCA: anti-neutrophil cytoplasmic antibody
C3: complement 3
*COL4A3*: Alpha 3 chain of collagen type IV
*COL4A4*: Alpha 4 chain of collagen type IV
*COL4A5*: Alpha 5 chain of collagen type IV
DMARDs: disease-modifying antirheumatic drugs
ESKD: End Stage Kidney Disease
ESR: erythrocyte sedimentation rate
GBM: glomerular basement membrane
IgA: Immunoglobulin A
IgG: Immunoglobulin G
IgM: Immunoglobulin M
MCP: metacarpophalangeal
NSAIDs: non-steroidal anti-inflammatory drugs
PIP: proximal interphalangeal
RA: rheumatoid arthritis
RF: rheumatoid factor
TBMN: thin basement membrane nephropathy

## References

[1] ashtan CE, Ding J, Garosi G, Heidet L, Massella L, Nakanishi K, Nozu K, Renieri A, Rheault M, Wang F, Gross O (2018). Alport syndrome: a unified classification of genetic disorders of collagen IV α345: a position paper of the Alport Syndrome Classification Working Group. Kidney Int.

[2] Yamamura T, Nozu K, Fu XJ, Nozu Y, Ye MJ, Shono A, Yamanouchi S, Minamikawa S, Morisada N, Nakanishi K, Shima Y, Yoshikawa N, Ninchoji T, Morioka I, Kaito H, Iijima K (2017). Natural history and genotype-phenotype correlation in female X-linked Alport syndrome. Kidney Int Rep.

[3] Cojocaru M, Cojocaru IM, Silosi I, Vrabie CD, Tanasescu R (2010). Extra-articular Manifestations in Rheumatoid Arthritis. Maedica (Buchar).

[4] Galesić K1, Prkacin I , Tisljar M, Vergles JM Renal involvement in patients with rheumatoid arthritis 2009;56(1):30–35. 20954306

[5] Kay J, Upchurch KS (2012). ACR/EULAR 2010 rheumatoid arthritis classification criteria. Rheumatology (Oxford).

[6] Makino H., Yoshinaga Y., Yamasaki Y., Morita Y., Hashimoto H., Yamamura M. (2002). Renal involvement in rheumatoid arthritis: analysis of renal biopsy specimens from 100 patients. Modern Rheumatology.

[7] Vinicki Juan P., Pellet Santiago C., De Rosa Graciela, Dubinsky Diana, Laborde Hugo A., Marini Alicia, Nasswetter Gustavo (2015). Analysis of 65 Renal Biopsies From Patients With Rheumatoid Arthritis (1976–2015). JCR: Journal of Clinical Rheumatology.

[8] Ohtani Hiroshi, Wakui Hideki, Komatsuda Atsushi, Okuyama Shin, Masai Rie, Maki Nobuki, Kigawa Akihiro, Sawada Ken-ichi (2004). Immune complex-type glomerulonephritis with unusual giant deposits in a patient with active rheumatoid arthritis. Clinical and Experimental Nephrology.

[9] Pierides A, Voskarides K, Kkolou M, Hadjigavriel M, Deltas C (2013). X-linked, COL4A5 hypomorphic Alport mutations such as G624D and P628L may only exhibit thin basement membrane nephropathy with microhematuria and late onset kidney failure. Hippokratia..

[10] Bekheirnia MR, Reed B, Gregory MC, McFann K, Shamshirsaz AA, Masoumi A, Schrier RW (2010). Genotype-phenotype correlation in X-linked Alport syndrome. J Am Soc Nephrol.

[11] Poduval P, Sillat T, Beklen A, Kouri VP, Virtanen I, Konttinen YT (2007). Type IV collagen alpha-chain composition in synovial lining from trauma patients and patients with rheumatoid arthritis. Arthritis Rheum.

[12] Gudmann NS, Junker P, Juhl P, Thudium CS, Siebuhr AS, Byrjalsen I, Karsdal MA, Bay-Jensen AC (2018). Type IV collagen metabolism is associated with disease activity, radiographic progression and response to tocilizumab in rheumatoid arthritis. Clin Exp Rheumatol.

[13] Abreu-Velez AM, Howard MS (2012). Collagen IV in normal skin and in pathological processes. N Am J Med Sci.

